# Noradrenergic control of error perseveration in medial prefrontal cortex

**DOI:** 10.3389/fnint.2012.00125

**Published:** 2013-01-02

**Authors:** Marcelo S. Caetano, Lu E. Jin, Linda Harenberg, Kimberly L. Stachenfeld, Amy F. T. Arnsten, Mark Laubach

**Affiliations:** ^1^Department of Neurobiology, Yale University School of MedicineNew Haven, CT, USA; ^2^The John B. Pierce LaboratoryNew Haven, CT, USA

**Keywords:** aging, behavioral flexibility, cognitive control, muscimol, noradrenaline, prefrontal cortex, working memory, yohimbine

## Abstract

The medial prefrontal cortex (mPFC) plays a key role in behavioral variability, action monitoring, and inhibitory control. The functional role of mPFC may change over the lifespan due to a number of aging-related issues, including dendritic regression, increased cAMP signaling, and reductions in the efficacy of neuromodulators to influence mPFC processing. A key neurotransmitter in mPFC is norepinephrine. Previous studies have reported aging-related changes in the sensitivity of mPFC-dependent tasks to noradrenergic agonist drugs, such as guanfacine. Here, we assessed the effects of yohimbine, an alpha-2 noradrenergic antagonist, in cohorts of younger and older rats in a classic test of spatial working memory (using a T-maze). Older rats (23–29 mo.) were impaired by a lower dose of yohimbine compared to younger animals (5–10 mo.). To determine if the drug acts on alpha-2 noradrenergic receptors in mPFC and if its effects are specific to memory-guided performance, we made infusions of yohimbine into mPFC of a cohort of young rats (6 mo.) using an operant delayed response task. The task involved testing rats in blocks of trials with memory- and stimulus-guided performance. Yohimbine selectively impaired memory-guided performance and was associated with error perseveration. Infusions of muscimol (a GABA-A agonist) at the same sites also selectively impaired memory-guided performance, but did not lead to error perseveration. Based on these results, we propose several potential interpretations for the role for the noradrenergic system in the performance of delayed response tasks, including the encoding of previous response locations, task rules (i.e., using a win-stay strategy instead of a win-shift strategy), and performance monitoring (e.g., prospective encoding of outcomes).

## Introduction

Medial parts of the prefrontal cortex (mPFC) have been implicated in the executive control of behavior. Lesions of mPFC result in disrupted instrumental learning (Corbit and Balleine, 2003), impairments in the inhibitory control of action (Passetti et al., 2002; Risterucci et al., 2003; Narayanan and Laubach, 2006), diminished flexibility following changes in the rules that govern action selection (Dias and Aggleton, 2000; Young and Shapiro, 2009), and deficits in performance adjustments after errors (Narayanan and Laubach, 2008). mPFC neurons become active when inappropriate or premature responses must be withheld (Narayanan and Laubach, 2006; Totah et al., 2009), when mistakes are made on prior trials (Narayanan and Laubach, 2008), and when action-reward contingencies are changed (Kargo et al., 2007; Rich and Shapiro, 2009). Based on these studies, mPFC seems crucial for two executive functions, inhibitory control, and behavioral flexibility.

Although the dopamine, serotonin, and noradrenaline (NA) systems have been implicated in executive functions [see Robbins and Roberts (2007) for review], the noradrenergic system in particular has been implicated in working memory (Arnsten and Goldman-Rakic, 1985; Wang et al., 2007), inhibitory control (e.g., Ma et al., 2003), and behavioral flexibility (e.g., Lapiz and Morilak, 2006). Studies using attentional set-shifting paradigms have shown that when the concentration of NA is reduced in PFC by lesioning the dorsal noradrenergic ascending bundle (DNAB) (Tait et al., 2007) or by manipulating alpha-2 NA autoreceptors (Lapiz and Morilak, 2006), the ability of rats to shift attention to a newly reward-relevant dimension in the environment is reduced. Theoretical studies have implicated the NA system within the mPFC in behavioral flexibility (Aston-Jones and Cohen, 2005; Bouret and Sara, 2005; Dayan and Yu, 2006). Recordings in the locus coeruleus during a signal-detection task found that NA neurons fire in a phasic manner when monkeys perform the task correctly and fire in a tonic manner when animals make mistakes (e.g., Clayton et al., 2004). Increased tonic activity by NA neurons leads to release of NA in the mPFC via the DNAB (Devauges and Sara, 1990). As such, blocking the ability of the NA system to regulate mPFC processing, e.g., using the selective alpha-2 antagonist yohimbine, should lead to error perseveration and a consequent impairments in behavioral flexibility. This hypothesis was tested in the present study using intra-cerebral infusions of yohimbine and muscimol during an operant delayed response task. The task was based on Horst and Laubach (2009, 2012), and was designed to assess memory and stimulus-guided responding in separate blocks of trials. Our findings suggest a role for the NA system within the mPFC in the control of error perseveration during memory-guided, but not stimulus-guided, performance.

The NA system may be especially important for cognitive control in aging. In both rats and monkeys, neurons in the frontal cortex exhibit significant anatomical alterations over the lifespan, such as dendritic regression (Grill and Riddle, 2002; Markham and Juraska, 2002; Duan et al., 2003; Dumitriu et al., 2010). These changes in neuronal complexity are accompanied by changes in NA in the mPFC, including reductions in projections from the locus coeruleus starting at 15 months (Ishida et al., 2000), decreases in NA synapses (Ishida et al., 2001a), and decreases in NA uptake, possibly due to the NA transporter and not to presynaptic autoreceptors (Shirokawa et al., 2003). Importantly, these changes are observed despite there being normal levels of NA in PFC in aged rats (Ishida et al., 2001b) and no major loss of cells in locus coeruleus in both aged human beings (Mouton et al., 1994) and rats (Goldman and Coleman, 1981). However, there are age-related decreases in alpha-2 receptors in the primate PFC, which may reflect reduced pre-synaptic receptors on NA synapses, but may also arise from reduced post-synaptic alpha-2 receptors on the spines of PFC pyramidal cells essential for working memory.

The persistent firing of PFC networks is needed for both working memory and behavioral inhibition (Funahashi et al., 1993). Post-synaptic alpha-2A receptors have an essential influence on PFC working memory circuits in rodents and primates, inhibiting cAMP-PKA opening of HCN (Hyperpolarization-activated Cyclic Nucleotide gated), and KCNQ channels to strengthen PFC network firing (Wang et al., 2007, 2011). In monkey PFC, alpha-2A receptors, HCN channels, and KCNQ channels have all been located on the post-synaptic membranes of pyramidal cell spines, next to glutamate, NMDA synapses (Aoki et al., 1998; Wang et al., 2007; Arnsten et al., 2012; Paspalas et al., 2012). Systemic or local stimulation of alpha-2A receptors with clonidine or guanfacine increases task-related firing during a working memory task, while blockade of these receptors with yohimbine markedly reduces PFC network firing (Li et al., 1999; Wang et al., 2007). Similar effects are seen on performance of a working memory task, where systemic or local administration of alpha-2 agonists improves working memory in rodents and primates, particularly in aged animals (Arnsten and Goldman-Rakic, 1985; Arnsten et al., 1988; Rama et al., 1996; Tanila et al., 1996). Conversely, systemic or local blockade of alpha-2 receptors with yohimbine impairs working memory performance (Arnsten and Goldman-Rakic, 1985; Li and Mei, 1994), as well as impulse control (Ma et al., 2003). Thus, loss of this key regulation with advancing age may contribute substantially to age-related cognitive decline. In aged monkey PFC, the reduction in persistent firing during working memory can be rescued by guanfacine (Wang et al., 2011). However, these data also suggest that the aged brain should also show altered sensitivity to NA receptor antagonists. To address this issue, we examined if there are age-related differences in the sensitivity of the mPFC to the alpha-2 antagonist yohimbine, using a standard T-maze task.

Together, our studies provide the first direct evidence for the noradrenergic system within the mPFC in the control of memory-guided performance and for aged animals being more sensitive to manipulations of noradrenergic processing.

## Materials and methods

All experimental procedures were approved by the Animal Care and Use Committee at Yale University (Study 1) and The John B. Pierce Laboratory (Study 2), and conform to guidelines for the Ethical Treatment of Animals (National Institutes of Health).

### Study 1, T-Maze delayed alternation task

#### Subjects

Nine aged (23–29-months old; Harlan) and seven young adult (5–10-months old; Taconic) male Sprague-Dawley rats were included in Study 1. They were extensively handled before training commenced. Throughout the behavioral training, all rats had unlimited access to water and regulated food access (16 g of standard chow per day).

#### Behavioral apparatus

**Figure 1:**
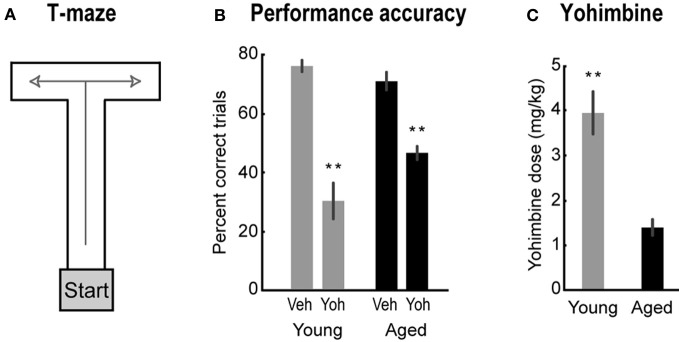
**(A)** The T-maze used in Study 1. **(B)** Percent correct for aged (black bars) and young rats (gray bars) after vehicle and yohimbine injections. Paired-samples *t*-test, Veh vs. Yoh, *t*_(8)_ = 5.93, *p* < 0.001 for aged rats; and *t*_(6)_ = 8.65, *p* < 0.001 for young rats. **(C)** Lowest dose of yohimbine to impair performance. One-Way ANOVA, *F*_(1, 14)_ = 29.88, *p* < 0.001. Error bars are SEM. ^**^Denotes *p* < 0.01.

Aged and young rats were trained in a delayed alternation task in a T-shaped maze (90 × 65 cm). The maze was composed of the start box at the bottom of the “T,” the center arm, and the right and left arms at the top of the “T” (Figure 1A). When the gate of starting box was lifted, the rat could move forward in the center arm and choose to enter the left or right arm to collect highly palatable miniature chocolate chips.

#### Training protocol

Rats were trained in a delayed alternation task based on methods described in Ramos et al. (2003). First, they were habituated to the T-maze until they were readily eating chocolate chips placed at the end of each arm. Then, training on the delayed alternation task commenced. In the first trial, animals were rewarded (i.e., collected the chocolate chips) for entering either arm, after which they were picked up and put back into the start box for a delay period. In the next 10 trials, rats were rewarded only if they entered the arm that was not chosen in the previous trial. During the delay period, the T-maze was wiped with alcohol to remove any olfactory clues left from the previous trial. Rats were tested for 1 session (11 trials) daily. The delay periods were adjusted for each rat to maintain performance at a stable baseline level (60–80% correct), ranging from 5 to 25 s. If they performed at 90–100% correct for two consecutive days, their delay periods were raised by 5 s.

#### Drug injections

When performance of rats trained in the T-maze was at baseline level for two consecutive days, they received an intraperitoneal mock injection (needle poke with no fluids injected) right before the daily session. If performance was at baseline level after the mock injection, then injections with yohimbine were administered in the following day.

Yohimbine hydrochloride (Tocris) was dissolved in sterile water at various concentrations. The drug was delivered systemically via intraperitoneal injections 20 min before testing. There was at least a week between each drug administration test session. Rats were first injected with vehicle (sterile water). If performance was still at baseline, then various doses of yohimbine (0.1–6 mg/kg) were tested as follows: each rat received a randomly chosen dose between 1 and 6 mg/kg. If the chosen dose caused impairment, the dose was lowered by 0.2–1 mg/kg until there was no impairment observed. As a result of this procedure, the number of doses differed over rats.

### Study 2, delayed response tasks

#### Subjects

Six young adult (6 months old at the start of training) male Brown Norway rats (Harlan) were maintained at ~85% of their free-feeding body weight with unlimited access to water and regulated food access throughout the behavioral training.

#### Behavioral apparatus

**Figure 2:**
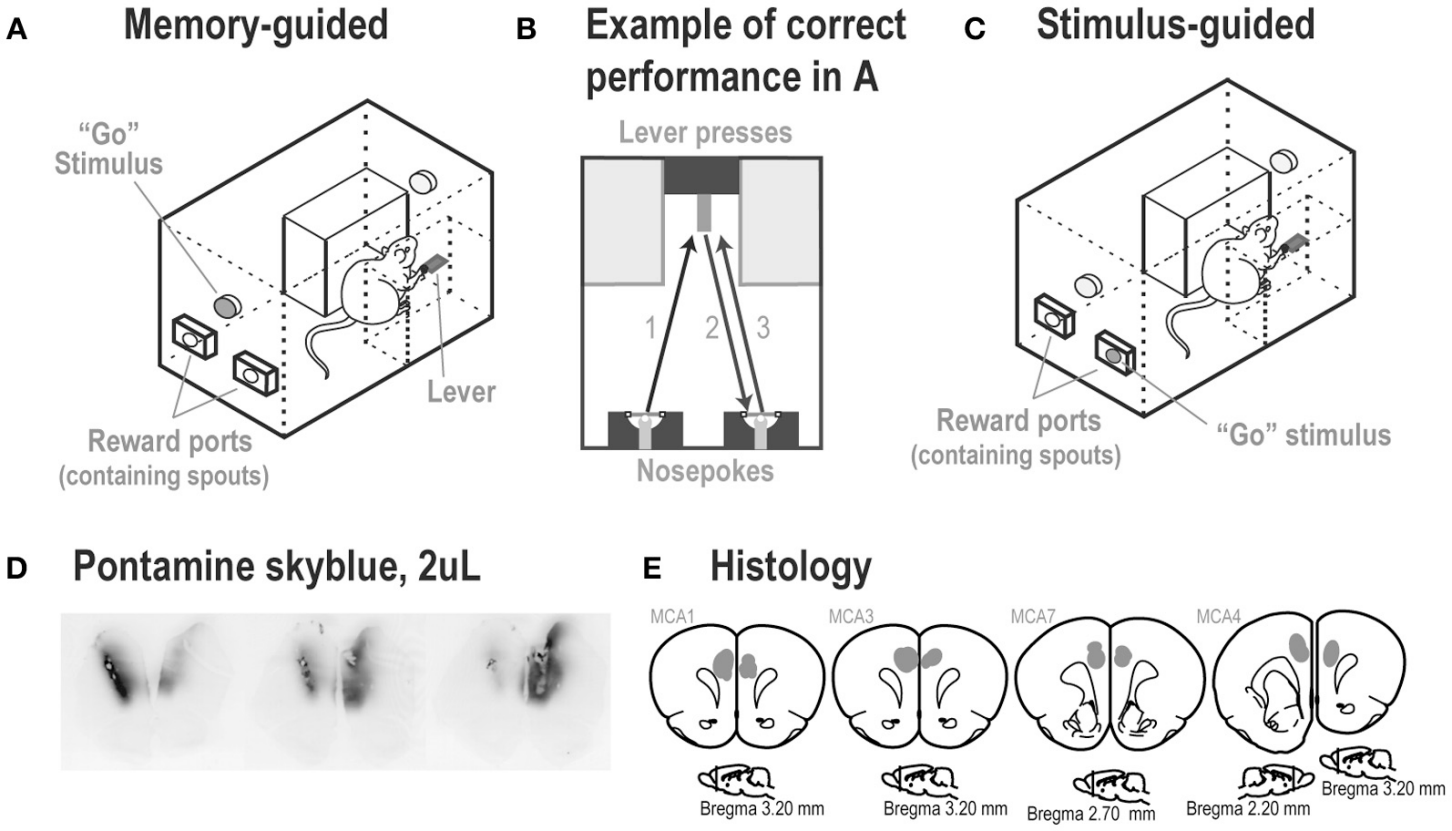
**(A)** Experimental box at the time of the Go stimulus and **(B)** performance of a correct trial in the memory-guided task. **(C)** A rat in the stimulus-guided task, in which a light inside one of the reward ports indicated the location for a correct head entry after a lever press. **(D)** Spread of 2 μL injections of the dye Pontamine sky blue into the mPFC of an untrained rat, shown over a series of ~50 μm thick sections. The center of the infusion in the left hemisphere is shown in the left brain section. The center of the infusion in the right hemisphere is shown in the right brain section. The center image was located between the two infusion sites. Note that the dye was retained within the medial prefrontal cortex. **(E)** Locations of infusion sites based on reconstructions of tissue dispersion patterns (shown as gray shaded areas) at the end of the tracks made by the guide cannula. In each rat, the cannula ended within the medial prefrontal cortex (Cg1/PrL areas). (Images are based on Paxinos and Watson, 1998).

Rats were trained in two delayed response tasks, based on methods described in Caetano et al. (2012). Standard operant boxes (ENV-008, Med Associates) equipped with a custom-made lever (The John B. Pierce Laboratory Instruments Shop), two diffuse houselights (ENV-215M, Med Associates), two head entry apertures (referred to as “reward ports”), and two LEDs located inside the reward ports (Figure 2) were used. The reward ports had spouts that delivered liquid sucrose and were equipped with infrared beams (ENV-114BM, Med Associates), which recorded times of head entries and licks to the spouts.

#### Surgeries

Before training, all rats in Study 2 were implanted bilaterally with 26-gauge 4 mm-long cannulae (Plastics One) into mPFC as described in Narayanan et al. (2006). Cannulae were targeted in mPFC at the following coordinates: AP: +3.2, ML: ±1.4, DV: −2.8 at 12° from the midline (See Figure 2E for locations of cannula tips).

#### Training protocols

***Memory-guided task.*** Rats were trained to perform an operant delayed alternation task as described in Caetano et al. (2012) over 20–25 sessions until they performed with an accuracy greater than 75% correct. Then, the rats were trained to perform a simple reaction-time task (using methods from Narayanan et al., 2006) and were shifted back to the delayed alternation task and experienced blocks of trials under memory and stimulus-guidance over 10–15 sessions. At this point, the rats were implanted with infusion cannulas and tested as described below.

A successful trial in the memory-guided task went as follows (Figure 2B): after a head entry into the left reward port, the light above the lever turned on and the rat moved to the lever (Figure 2B, arrow 1). The first lever press delivered ~0.05 ml of 20% sucrose solution at the spout above the lever, turned off the light above the lever, and turned on the light above the reward ports (Go stimulus). After choosing the correct (alternate) reward port (Figure 2B, arrow 2), the light above the reward ports turned off, the light above the lever turned on, and ~0.05 ml of 20% sucrose solution was delivered from the spout inside the correct port. If the rat chose the same port from the previous trial (incorrect trial), no liquid sucrose was delivered and the trial restarted. After incorrect trials, the same reward port assignment was kept for the next trial (correction trial). Rats were trained in 60-min daily sessions (excluding weekends).

***Stimulus-guided task.*** The stimulus-guided task was similar to the memory-guided procedure, except that the Go stimulus was presented inside the left or right reward port (determined randomly across trials and with no correction trials), and its location defined the assigned correct port for a head entry in each trial (Figure 2C). Therefore, the rats had to break from the “alternation mode” and “track the stimulus” to receive the reward.

When performance was >75% correct in the stimulus-guided task, all rats were exposed to the two tasks within the same training session. Each session started with a block of memory or stimulus-guided trials (counterbalanced across rats), and switched every time the rats completed 40 correct trials. After two 60-min sessions on this mixed protocol, the testing sessions commenced. Rats experienced 10–15 sessions of training in the stimulus-guided task and sessions with shifting versions of the two tasks.

#### Drug infusions

The goal of this study was to assess the effects of blocking alpha-2 NA receptors, using yohimbine, in the mPFC during the operant delayed response task. The dose, volume, and timing for testing were based on the study by Ma et al. (2003). After we found effects of yohimbine on the animals' performance of the task, we tested the animals with infusions of muscimol (to carry out reversible inactivation) to determine if cortical sites that were sensitive to alpha-2 blockade were also sensitive to reversible inactivation. For this reason, we did not counterbalance the order of drug testing and did not test a wide range of doses of yohimbine.

Over three consecutive days, each rat received a bilateral infusion of saline (day 1), yohimbine (day 2), and a recovery session with no infusions (day 3). Rats were run on the mixed protocol with the same order of counterbalancing of initial tasks across all sessions. At the end of the experiment all rats received bilateral infusions of muscimol and a second recovery session.

Infusions were done as described in Narayanan et al. (2006). Rats were briefly anesthetized with 4% isoflurane to ensure a low level of stress and a high level of consistency across infusions (testing done in sessions prior to infusions showed no effects of brief anesthesia in these tasks). A total volume of 2 μl of sterile saline (0.9%; Phoenix Scientific) and yohimbine hydrochloride (5 μg/μL) (Tocris) dissolved in saline were delivered bilaterally at a rate of 0.5 μL/min. The spread of a 2 μl infusion into mPFC was assessed in an untrained rat with 1% pontamine sky blue (following the protocol by Li and Mei, 1994), and was sufficient to cover most of—but was confined to—the target region (mPFC) (Figure 2D). At the end of the experiment, 0.5 μl of muscimol (0.05 μg/μL) (Tocris) dissolved in saline was delivered bilaterally at a rate of 0.25 μl/min. Previous studies have used this volume and rate of fluorescent muscimol infusions to confirm the approximate extent of cortical inactivation in mPFC, which is estimated at approximately 1 mm^2^ (Narayanan et al., 2006; Allen et al., 2008). Rats were tested 15 min after infusion of saline and yohimbine (Li and Mei, 1994), and 45 min after infusion of muscimol to allow maximal inhibition of cortical activity (Martin and Ghez, 1999; Allen et al., 2008). Please note that given differences in the volumes infused for yohimbine and muscimol and the estimates of spread made using dye (Figure 2D), the effects of muscimol were likely to occur over a smaller total cortical volume that than obtained with yohimbine.

#### Histology

Once experiments were complete, rats in Study 2 were anesthetized with 1 ml Euthasol and transcardially perfused with 10% formalin. Brains were later sectioned, mounted, and stained for Nissl with thionin using standard methods.

Cannula tracts were based on microscopic assessment of thionin-stained sections, made using a Motic microscope and a BioQuant computer-assisted reconstruction system. Drug injection sites were estimated based on cannula tracts and regions with tissue dispersion at the tips of the cannula tracts. Sections with evidence for cannula tracts and tissue dispersion at the tips of the tracts were mapped onto standard atlas sections (Paxinos and Watson, 1998).

Two of six implanted rats were excluded from the study. One rat did not have both guide cannulas in the mPFC. One cannula was implanted into the most rostral medial frontal cortex and the other appeared to have destroyed the medial frontal cortex along the edge of the hemisphere. Infusions on this side likely went directly into the brain case. Another rat appeared to have blocked cannulas and it was not possible to infuse any drugs in this animal.

#### Data analysis

The number of trials completed in the 60 min testing sessions differed across the three testing sessions (One-Way ANOVA, *F*_(2, 6)_ = 5.26, *p* < 0.05). Mean number of trials (and SEM) were 169.3 (±36) for saline, 222.5 (±21.6) for yohimbine, and 221 (±20.5) for recovery sessions. Therefore, to standardize the number of trials analyzed across subjects and conditions, only the first two blocks of trials per session were used (i.e., two rats with a block of memory-guided trials followed by a block of stimulus-guided trials, and two rats with stimulus- followed by memory-guided trials). Performance in the muscimol session was compared with performance in the immediately following recovery session.

## Results

### Study 1—age-related impairment of yohimbine on spatial working memory performance

Systemic injections of yohimbine significantly impaired performance in the T-maze for both young and aged rats. After yohimbine injections, percent correct trials decreased for both groups of rats compared to performance after vehicle injections [Figure 1B; paired-samples *t*-test, Veh vs. Yoh, *t*_(8)_ = 5.93, *p* < 0.001 for aged rats; and *t*_(6)_ = 8.65, *p* < 0.001 for young rats]. Performance in young rats under yohimbine was below chance (50% correct; Figure 1B), a finding that suggests yohimbine may cause a transition from rule-guided alternations to perseverative actions. This hypothesis was tested in the second part in this study. Interestingly, aged rats were more sensitive to the effects of yohimbine than younger rats. The lowest doses that impaired performance were significantly lower in aged rats compared to young rats [Figure 1C; One-Way ANOVA, *F*_(1, 14)_ = 29.88, *p* = 0.001]. To assess if the higher effective dose in young rats was due to the more severe impairment found in the younger animals, we compared the slightly lower doses which did not cause impairments in young rats with the impairment-producing doses in aged rats, and found the difference still significant [One-Way ANOVA, *F*_(1, 14)_ = 18.34, *p* < 0.001, data not shown]. These results suggest that lower doses are required to impair performance in aged animals.

### Study 2—noradrenergic effects on performance accuracy in the memory- and stimulus-guided tasks

**Figure 3:**
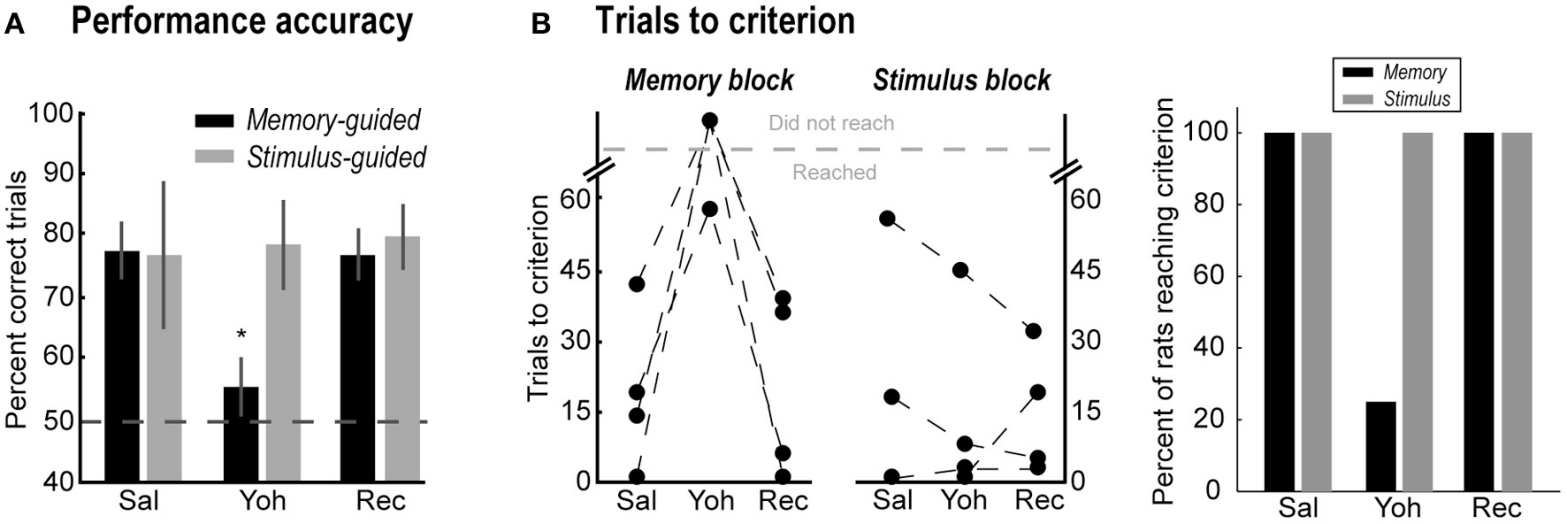
**(A)** Percent correct trials in the memory-guided (black bars) and stimulus-guided (gray bars) tasks after saline (Sal), yohimbine (Yoh), and in a recovery session (Rec). Error bars are SEM. ^*^Denotes *p* < 0.05. **(B)** Trials to reach the performance criterion in the memory-guided trials (left panel) and in the stimulus-guided trials (right panel), and percent of rats that reached the criterion (rightmost panel).

Yohimbine infusions selectively decreased accuracy in the memory-guided task (Figure 3A, black bars) compared to the saline session, and rebounded to control levels in the recovery session [One-Way ANOVA, *F*_(2, 6)_ = 9.13, *p* = 0.015; paired-samples *t*-test: Sal vs. Yoh, *t*_(3)_ = 8.49, *p* = 0.003; Yoh vs. Rec, *t*_(3)_ = 3.38, *p* = 0.043]. Yohimbine infusions had no effect in accuracy during stimulus-guided trials [gray bars; One-Way ANOVA, *F*_(2, 4)_ = 0.65, *p* = 0.569]. One rat (Rat 2) was excluded from the analysis of percent correct in the stimulus-guided task because after reaching 40 correct trials in the initial memory-guided task after saline infusion, it completed only a few trials in the stimulus-guided task. This rat, however, completed 172 and 169 overall trials in the yohimbine and recovery sessions, respectively, with >75% correct accuracy in the stimulus-guided trials.

Figure 3B shows the effects of yohimbine on the number of trials required to reach the performance criterion in each block of trials (left panels), defined as 90% correct performance over a block of 10 consecutive trials, and the percent of rats that reached this criterion (rightmost panel). After yohimbine infusions, one rat required many more trials to reach that criterion in the memory-guided block compared to the saline session (Rat 2, from 19 to 58 trials), while the remaining three rats did not reach the criterion at all. In contrast, all rats reached the performance criterion in the stimulus-guided task in all testing sessions (Figure 3B, right plot; gray bars in rightmost panel). Note that yohimbine consistently disrupted performance in the memory-guided trials regardless of whether this block of trials was the first or second in the session (counterbalanced across rats).

### Noradrenergic effects on error perseveration

**Figure 4:**
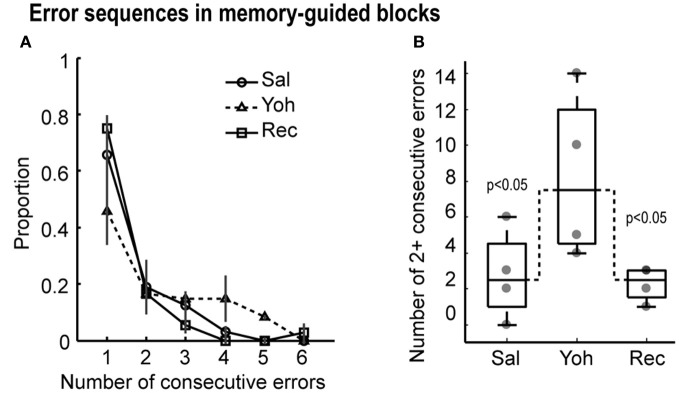
**(A)** Proportion of consecutive errors in the memory-guided task. Note that the proportion of sequential errors increased under yohimbine, leading to a lower overall proportion of isolated errrors. **(B)** Boxplots with the number of two or more consecutive errors in the testing sessions. Gray dots depict the values for each of the four rats. Dashed lines link the median values for more than two consecutive errors, which were significantly different by Friedman's ANOVA at *p* < 0.05.

The distribution of errors after yohimbine infusions differed from the distribution of errors during saline infusions (Friedman's ANOVA, *X*^2^ = 4.3, *p* = 0.038) and during recovery (Friedman's ANOVA, *X*^2^ = 7.1, *p* = 0.008) in memory-guided trials (Figure 4A). Specifically, the number of two or more consecutive errors increased in the yohimbine session (Figure 4B). These results suggest that mPFC infusions of yohimbine impaired the ability of rats to adjust performance after mistakes.

### Muscimol reduced accuracy in the memory blocks but did not lead to perseveration

**Figure 5:**
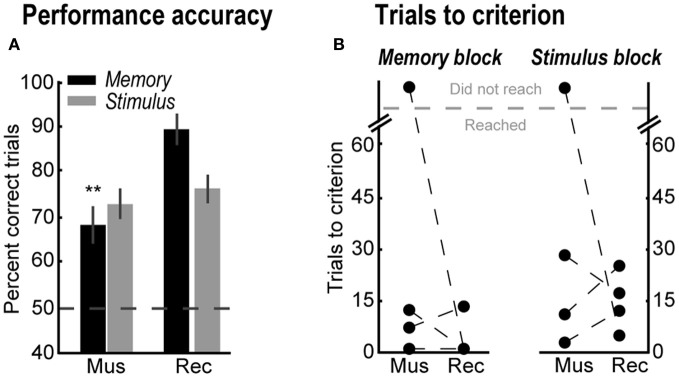
**(A)** Percent correct trials in the memory-guided (black bars) and stimulus-guided (gray bars) tasks after muscimol infusions (Mus), and in a recovery session (Rec). Error bars are SEM. ^**^Denotes *p* < 0.01. **(B)** Trials to reach the performance criterion in the memory- and stimulus-guided trails after muscimol infusions (Mus) and in the recovery session (Rec).

In order to confirm the involvement of the yohimbine-sensitive sites in spatial working memory performance, mPFC was inactivated with bilateral infusions of muscimol. Compared with the following recovery session, muscimol infusions decreased percent correct trials in the memory-guided task [Figure 5A, black bars; Paired-samples *t*-test, *t*_(3)_ = 11.37, *p* = 0.002], but not in the stimulus-guided task [Figure 5A, gray bars; Paired-samples *t*-test, *t*_(3)_ = 0.72, *p* = 0.523]. In contrast to yohimbine infusions, after muscimol infusions only one rat was unable to reach the performance criterion in the memory-guided task (Rat 2) or in the stimulus-guided task (Rat 4) (Figure 5B).

### Lack of effects of yohimbine and muscimol infusions on temporal aspects of the tasks

**Figure 6:**
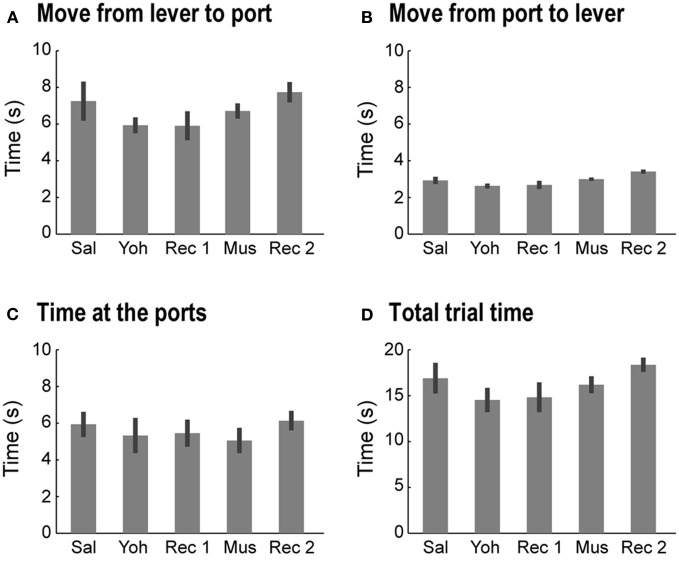
**Non-specific effects of mPFC infusions of yohimbine.** Yohimbine and muscimol infusions did not alter the time spent in each task component or the time to transition between components (One-Way ANOVAs: *p* >> 0.05, for all measures). **(A)** Average (and SEM) time spent to move from the lever to the reward ports in the 5 testing sessions. **(B)** Average (and SEM) time spent to move from the reward ports to the lever in the 5 testing sessions. **(C)** Average (and SEM) time spent at the reward ports in the 5 testing sessions. **(D)** Average (and SEM) time spent completing one trial in the 5 testing sessions.

Yohimbine and muscimol infusions did not alter the time spent by the rats at the lever or at the reward ports, the time to transition between those components (move to/from the ports), or the total trial time in either task (One-Way ANOVA: *p* >> 0.05 for all measures; Figure 6). When data from the two tasks were collapsed, no consistent effect of either drug was found in any of the temporal measures or in the proportion of trials in which the rats checked both reward ports within the same trial. These null results suggest that neither drug increased impulsivity in the tasks.

## Discussion

In Study 1, older rats were impaired on the T-maze by lower doses of yohimbine compared to younger animals. In Study 2, performance was impaired by infusions of yohimbine into mPFC during the memory-guided, but not the stimulus-guided, task. Rats infused with yohimbine made more consecutive errors when performing based on memory, suggesting that their ability to correct performance after mistakes was diminished. These results implicate the NA system within mPFC in memory-specific behavioral adjustments. To our knowledge, the present study is the first report of such memory-specific effects of the NA system within the rodent mPFC.

Yohimbine has also been shown to impair behavioral performance in a variety of species and paradigms. In Ma et al. (2003), for example, monkeys subjected to prolonged infusion of yohimbine into PFC were unable to withhold responding in a go/no-go task. PFC infusions of yohimbine also impaired performance in working memory tasks in monkeys, such as the delayed-response task (Li and Mei, 1994; Li et al., 1999) and the oculomotor delay-response task (Sawaguchi, 1998). In rats, intraperitoneal injections of yohimbine increased premature responding in a differential reinforcement of low response rate (DRL) schedule (Sanger, 1988). Moreover, administration of alpha-2 NA agonists, such as guanfacine or clonidine, has been shown to enhance performance in spatial memory tasks (Li et al., 1999; Wang et al., 2011).

Despite these reports, opposing effects have been observed in rats tested under alpha-2 NA antagonists in other paradigms, supporting the view that manipulations of monoamines within PFC may have task-specific effects (Arnsten, 1998). For example, monoamine depletions in PFC impair spatial working memory performance (e.g., Collins et al., 1998) but not performance on set-shifting tasks (e.g., Roberts et al., 1994). Studies that manipulated the NA system, in particular, support the notion of task-dependent effects. Increasing the concentration of NA in mPFC via intraperitoneal injections of idazoxan (Devauges and Sara, 1990), and mPFC infusions of atipamezole (Lapiz and Morilak, 2006), both of which are alpha-2 NA antagonists, have been shown to enhance cognitive performance in attentional set-shifting tasks. When NA is depleted in mPFC through lesions of the DNAB, cognitive performance in those tasks is impaired (Tait et al., 2007). There are at least three plausible interpretations of these findings. First, alpha-2 NA antagonists improve performance in tasks that involve stimulus-outcome associations, such as attentional set-shifting tasks, but impair performance in tasks that rely on action-outcome associations, such as spatial tasks. Second, alpha-2 NA antagonists affect aspects of temporal order processing that are involved in delayed response alternation tasks but not in stimulus-tracking tasks. Third, alpha-2 receptor stimulation helps to maintain an attentional set through increased persistent firing, and these physiological actions which strengthen working memory also interfere with the ability to shift attentional set. New studies are needed to resolve these issues.

In Study 2, percent correct in the stimulus-guided task was unaffected by the yohimbine infusions, both at the beginning of the session and after a block of memory-guided trials. The stimulus-guided task involved the same set of movements and actions used in the memory-guided task, but instead of relying on internal cues (reward port visited in the previous trial) rats had to rely on an external cue (location of the Go stimulus). In a related stimulus-guided task, Sun et al. (2010) also reported no effects of intraperitoneal injections of yohimbine in accuracy. Instead, the authors observed an increase in impulsivity in the rats, indicated by an increase in premature entries into the apertures. An increase in premature responding has also been observed in response to muscimol inactivation of mPFC in rats trained in a simple reaction time task (e.g., Narayanan et al., 2006) and in response to intraperitoneal injections of yohimbine in rats trained in a DRL schedule (Sanger, 1988). These results are in agreement with the idea that PFC exerts inhibitory control of actions with a possible involvement of the noradrenergic system.

In the present study, reversible inactivations of mPFC with muscimol led to impairment only in the memory-guided task, which confirms the involvement of mPFC in spatial working memory performance (Horst and Laubach, 2009) and strategy switching (Rich and Shapiro, 2009). The effects of muscimol reported in this study are less dramatic compared to another study from our lab (Horst and Laubach, 2009). A key difference between these studies is that the interval between responses in the present study was only a few seconds, much shorter than the delays used in Horst and Laubach (2009). In the present study, inactivation of mPFC did not alter the number of trials required for rats to reach the performance criterion (Figure 5B). Likewise, in our previous study we found no evidence for error perseveration following inactivation of mPFC during a standard operant delayed alternation task (Horst and Laubach, 2009). Therefore, we propose that our present results suggest that blocking a single modulatory input to mPFC (alpha-2 noradrenergic receptors) leads to a specific change in performance that is distinct from inhibiting processing within the cortical region.

The effects of the alpha-2 NA antagonist could be due to changes in the excitability of mPFC networks (Ji et al., 2008; Mueller et al., 2008), an effect that is opposite to muscimol (van Duuren et al., 2007). However, both the Ji and Mueller studies did not assess mPFC activity in tasks that are known to depend on mPFC processing, as in the types of delayed response tasks that are used in the present study. A plausible hypothesis for the actions of yohimbine in mPFC is that it disrupts normal patterns of network activity that may occur in advance of errors, as found in a recent human EEG study (Cavanagh et al., 2009). If the same process exists in rats and is sensitive to manipulations of the NA system, then our rats would be unable to monitor behavioral outcomes and adjust performance when mistakes are likely to be made. Neuronal evidence for such prospective coding of behavioral outcomes has been reported during an mPFC-dependent simple reaction-time task (Narayanan and Laubach, 2006) and two types of spatially delayed response tasks (Hyman et al., 2011, 2012; Horst and Laubach, 2012).

This mechanism of action would be different from a total inactivation of mPFC and may explain how blocking a single modulatory input to mPFC (alpha-2 noradrenergic receptors) would lead to a specific change in performance that is distinct from effects of a more general inhibition by muscimol. Along these lines, electrophysiological studies in rats (e.g., Bouret and Sara, 2004) and non-human primates (Clayton et al., 2004; Aston-Jones and Cohen, 2005) have suggested the involvement of the NA system in behavioral flexibility, and computational studies have proposed that the activity of noradrenergic neurons within mPFC may serve as a network reset signal that correlates with behavioral flexibility and arises when behavior adjustment is needed (Bouret and Sara, 2005; Dayan and Yu, 2006). Several recent studies have reported evidence for the cross-trial encoding of behavioral outcomes in the frontal cortex during non-spatial (e.g., Seo and Lee, 2007; Narayanan and Laubach, 2008; Quilodran et al., 2008; Histed et al., 2009; Totah et al., 2009) and spatial (Host and Laubach, 2012; Hyman et al., 2012) tasks. It is possible that neural activity associated with these cross-trial encodings of behavioral outcome depends on the alpha-2 NA system. Resolving this issue should be a future direction of research on the mPFC.

### Conflict of interest statement

Dr. Amy F. T. Arnsten and Yale University have a license agreement and receive royalties from Shire Pharmaceuticals for the sale of Intuniv™ (extended release guanfacine) for the treatment of pediatric ADHD. The other authors declare that the research was conducted in the absence of any commercial or financial relationships that could be construed as a potential conflict of interest.
