# Erratum: Noradrenergic control of error perseveration in medial prefrontal cortex

**DOI:** 10.3389/fnint.2013.00048

**Published:** 2013-06-26

**Authors:** Mark Laubach

**Affiliations:** The John B. Pierce LaboratoryNew Haven, CT, USA

The paper should have listed support from the National Science Foundation (NSF). Two authors (Stachenfeld and Laubach) were supported with funds from NSF grant 1121147 to Mark Laubach.

The correct acknowledgment is:

“This work was supported by Public Health Service Grant PO1AG030004 from the National Institute on Aging (Amy F. T. Arnsten and Mark Laubach), the National Science Foundation 1121147 (Mark Laubach) and by AFAR/Ellison Medical Foundation Post-doctoral Fellowship (Marcelo S. Caetano).”

